# Cranberry-derived proanthocyanidins induce a differential transcriptomic response within *Candida albicans* urinary biofilms

**DOI:** 10.1371/journal.pone.0201969

**Published:** 2018-08-08

**Authors:** Anitha Sundararajan, Hallie S. Rane, Thiruvarangan Ramaraj, Johnny Sena, Amy B. Howell, Stella M. Bernardo, Faye D. Schilkey, Samuel A. Lee

**Affiliations:** 1 National Center for Genome Resources, Santa Fe, NM, United States of America; 2 Section of Infectious Diseases, New Mexico VA Healthcare System, Albuquerque, NM, United States of America; 3 Marucci Center for Blueberry and Cranberry Research and Extension, Rutgers, The State University of New Jersey, Chatsworth, NJ, United States of America; 4 Division of Infectious Diseases, University of New Mexico Health Science Center, Albuquerque, NM, United States of America; Leibniz-Institut fur Naturstoff-Forschung und Infektionsbiologie eV Hans-Knoll-Institut, GERMANY

## Abstract

*Candida albicans* is one of the most common causes of hospital-acquired urinary tract infections (UTIs). However, azoles are poorly active against biofilms, echinocandins do not achieve clinically useful urinary concentrations, and amphotericin B exhibits severe toxicities. Thus, novel strategies are needed to prevent *Candida* UTIs, which are often associated with urinary catheter biofilms. We previously demonstrated that cranberry-derived proanthocyanidins (PACs) prevent *C*. *albicans* biofilm formation in an *in vitro* urinary model. To elucidate functional pathways unique to urinary biofilm development and PAC inhibition, we investigated the transcriptome of *C*. *albicans* in artificial urine (AU), with and without PACs. *C*. *albicans* biofilm and planktonic cells were cultivated with or without PACs. Genome-wide expression analysis was performed by RNA sequencing. Differentially expressed genes were determined using DESeq2 software; pathway analysis was performed using Cytoscape. Approximately 2,341 of 6,444 total genes were significantly expressed in biofilm relative to planktonic cells. Functional pathway analysis revealed that genes involved in filamentation, adhesion, drug response and transport were up-regulated in urinary biofilms. Genes involved in carbon and nitrogen metabolism and nutrient response were down-regulated. In PAC-treated urinary biofilms compared to untreated control biofilms, 557 of 6,444 genes had significant changes in gene expression. Genes downregulated in PAC-treated biofilms were implicated in iron starvation and adhesion pathways. Although urinary biofilms share key features with biofilms formed in other environments, many genes are uniquely expressed in urinary biofilms. Cranberry-derived PACs interfere with the expression of iron acquisition and adhesion genes within urinary biofilms.

## Introduction

*Candida albicans* and related species are the third leading cause of hospital-associated urinary tract infections (UTIs), and are a marker of increased mortality and healthcare costs [1–4]. *Candida* UTIs disproportionately impact ICU patients, burn patients, neonates, and those with immunosuppression, malignancy, diabetes, and urologic abnormalities [5,6]. Most cases are associated with the use of urinary catheters [7]. Of note, the Centers for Medicare and Medicaid Services are no longer reimbursing healthcare systems for hospital-acquired UTIs. Although three major classes of antifungal therapy are available, each has important limitations. Echinocandins do not achieve effective urinary concentrations, lipid amphotericin causes substantial renal and other toxicities, and fluconazole can be limited by drug resistance, particularly within biofilms. Furthermore, urinary concentrations of posaconazole, voriconazole, and itraconazole are very low [8]. Our understanding of *C*. *albicans* urinary pathogenesis is surprisingly limited [8,9], and there is a lack of strategies to prevent development of *Candida* UTIs [10]. Thus, discovery of effective methods to prevent *Candida* UTIs, based on a greater understanding of *Candida* urinary molecular pathogenesis, would represent an important advance.

*C*. *albicans* readily forms biofilms on both biotic and abiotic surfaces, a major attribute that contributes to pathogenesis [11,12]. Although less well understood than biofilms formed in intravascular catheters, urinary biofilms are a key component of urinary pathogenesis [13–15]. As demonstrated in a rat urinary catheter model, *C*. *albicans* urinary biofilm formation occurs over 24–48 h, followed by pyuria, acute cystitis, and bladder tissue invasion with hyphae [16]. The initial stage of biofilm formation relies on adhesion, which is dependent on both non-specific bio-physical interactions and specific expression of adhesins and other cell-surface proteins [17]. Interestingly, a *C*. *albicans* adhesion mutant lacking the adhesins Als1p and Als3p was less virulent in this rat urinary catheter model [16]. Therefore, inhibition of adherence by *C*. *albicans* is one attractive strategy for disrupting the initial stages of urinary biofilm formation.

While short and long-term urinary catheters are still overused in clinical practice, in many instances they are unavoidable (e.g. trauma, urinary obstruction, spinal cord injury, etc.). Because urinary catheter flow needs to be maintained, antimicrobial lock therapies are not feasible. Silver-impregnated and other coated urinary catheters have been used for two decades, and while they have reduced the short-term incidence of asymptomatic bacteriuria, they have proven to be of marginal (or no) value for prevention of catheter-associated UTIs [18]. In contrast, there is an extensive body of literature demonstrating the preventative value of cranberry juice against *Escherichia coli* UTIs [19–22]. Anti-adherence and iron chelation mechanisms of cranberry juice extract against *E*. *coli* are well-described [23–29]. Cranberry-derived proanthocyanidins (PACs) exhibit both non-biospecific activity against adherence [25], and biospecific activity, including reduced expression of *E*. *coli* adhesion genes [27]. PACs also reduce *E*. *coli* adherence to synthetic materials such as PVC and polytetrafluoroethylene [25]. Cranberry PACs decrease *C*. *albicans* adherence to oral epithelial cells and reduce biofilm formation and inflammatory responses *in vitro* [30]. Furthermore, PACs have been shown to cause iron starvation in uropathogenic *E*. *coli* by iron chelation [28,29]. These studies highlight the potential of cranberry PACs for prevention of UTIs via inhibition of adherence and iron acquisition.

In contrast, there is extremely limited data on the role of cranberry juice and PACs for the prevention of *Candida* UTIs. Further, there is little specific data on the molecular pathogenesis of urinary biofilm formation, candiduria, and infection of the urinary tract. We have previously demonstrated that cranberry PACs prevent the development of *C*. *albicans* urinary biofilms formed on polystyrene or silicone in an *in vitro* model of biofilm formation in artificial urine (AU) [31]. Mechanistically, we have shown that PACs inhibit *C*. *albicans* adhesion, and that iron supplementation partially reverses inhibition of biofilm formation [31]. In these experiments, we utilized genome-wide transcriptional analysis to further define the mechanism of action of cranberry PACs, and gain more understanding of features unique to *C*. *albicans* biofilm formation in an artificial urinary environment.

## Materials and Methods

### Strains, media, and reagents

Cranberry PACs were isolated from cranberry fruit (*Vaccinium macrocarpon* Ait.) using solid-phase chromatography as previously described [23,32,33]. The cranberry PACs were derived from a mixture of the cranberry varieties ‘Stevens’ and ‘Early Black’ grown in New Jersey at the Marucci Center for Blueberry Cranberry Research, Rutgers University, New Jersey, USA. All experiments were completed using *C*. *albicans* strain SC5314 (gift from W. Fonzi, Georgetown University, Washington DC, USA) [34]. Initial cell cultures were grown in YPD (1% yeast extract, 2% peptone, and 2% glucose), supplemented with 80 μg/mL uridine to promote better growth. Artificial urine (AU, a defined medium composed of 0.65 g/L CaCl_2_, 0.65 g/L MgCl_2_, 4.6 g/L NaCl, 2.3 g/L Na_2_SO4, 0.65 g/L sodium citrate, 0.02g/L sodium oxalate, 2.8 g/L KH_2_PO_4_, 1.6 g/L KCl; 1.0 g/L NH_4_Cl, 25.0 g/L urea, 1.1 g/L creatinine, 0.34 g/L YNB without amino acids, 0.04 g/L CSM-URA, 80 g/L D-glucose, and 80 μg/mL uridine) was prepared using a published recipe [13].

### Treatment conditions and RNA extraction

Cells were grown under four separate conditions: planktonic (or free-living) cells, planktonic cells treated with 256 μg/mL cranberry PACs, biofilm cells, and biofilm cells treated with 256 μg/mL cranberry PACs. The concentration of 256 μg/mL was picked as it is the lowest dose with a significant effect on both biofilm formation and biofilm treatment [31]. For each condition, RNA was extracted from three separate biological replicates. Biofilm formation was completed using a previously published static microplate model [35] with several modifications. Firstly, biofilms were formed in six-well polystyrene plates rather than 96-well plates to increase RNA yield. Secondly, biofilms were grown for 48h rather than 24h to allow artificial urine biofilms to fully mature, as previously described [13]. Preliminary experiments were conducted to determine the optimal cell concentration and volume in 6-well plates to maximize biofilm formation; a starting cell inoculum of 5×10^5^ cells/mL and a volume of 6 mL per well generated the most robust biofilms. Using the optimized inoculum, planktonic and biofilm cells were prepared for RNA extraction. Briefly, *C*. *albicans* SC5314 cells from overnight cultures in YPD were washed three times in 1XPBS and added to AU ± 256 μg/mL cranberry PACs to a concentration of 5×10^5^ cells/mL. For planktonic treatments, 5 mL of cells in AU ± cranberry PACs were grown in a 50mL conical tube at 30°C, 250 rpm for 48h. For biofilm treatments, a 6 mL volume of cells in AU ± cranberry PACs was added to the wells of a six-well plate; the plate was incubated in a 37°C static incubator for 48h. After the 48h incubation, AU was aspirated from the biofilm wells via pipetting. Biofilm cells were collected by adding 1mL ice-cold RNase-free water (Life Technologies) to each well and scraping adherent cells from the bottom of each well. The scrape-collection step was repeated a second time to ensure full collection of biofilm cells. Collected biofilm cells in ice-cold water were pelleted via centrifugation at 250×g, 4°C for ten minutes. RNA was extracted from both biofilm and planktonic treatments ± cranberry PACs using the Ambion RiboPure™ yeast RNA Purification Kit (ThermoFisher Scientific) according to the manufacturer’s protocol.

### RNA-sequencing

RNA libraries were prepared using standard Illumina TruSeq library kits. A PolyA selection step was performed as part of the library protocol to enrich for messenger RNA. Prepared libraries were then sequenced on an Illumina HiSeq 2000 instrument to generate 50nt single end reads. Raw sequence reads were post-processed to remove Illumina adapters/primer sequences. Sequence data generated has been deposited at NCBI under bioproject number PRJNA338054.

### Transcriptome analysis

Post-processed high quality reads for each sample were aligned to *C*. *albicans* SC5314 Assembly 22 using GSNAP (version released on 2014_12_29) with indel penalty set to 2, maximum mismatches set to 0.06 and all other parameters set to default [36]. Read counts were generated using the Alpheus pipeline developed at NCGR [37]. Gene expression was quantified as the total number of reads for each sample that uniquely aligned to the reference, binned by annotated gene coordinate. Annotation file in GFF format associated with the assembly in this study was used for gene coordinate information and to bin the reads. *C*. *albicans* is a diploid organism; since both haplotypes were present in the assembly file, read counts for each gene were computed by adding all the multi-mapping reads for both alleles of the gene. Differential expression and related quality control analyses was investigated using the Bioconductor package, DESeq2 [38] with the DEApp tool [39]. Raw gene read-count values were normalized for differences in sequencing depth and composition using strategies implemented in DESeq2, enabling gene expression comparisons across samples. Differential expression analysis of the per-sample, normalized read counts were assessed with the negative binomial test as implemented in DESeq2 with the Benjamini-Hochberg false discovery rate (FDR) adjustment [40] applied for multiple testing corrections. For our analysis, a false discovery rate of 0.05 was applied and any candidate with a p-adjusted value of less than or equal to 0.05 and a fold-change of 1.5 or higher was defined as significantly up- or down- regulated.

Venny (*http*:*//bioinfogp*.*cnb*.*csic*.*es/tools/venny/index*.*html*), an online Venn diagram tool, was used to identify shared differentially-expressed genes between the three treatments as compared to planktonic cells in AU, namely, control biofilm, PAC-treated biofilms and PAC-treated planktonic cells [41].

The top differentially-expressed genes across all treatments as compared to planktonic cells in AU were selected using DESeq2 with the DEApp tool, with an increased cut-off defined as a p-adjusted value of less than or equal to 0.01 and a fold-change of 5 or higher. The top differentially-expressed genes were visualized using the Heatmapper web tool [42].

### Pathway analysis

Biological information in the context of pathways was obtained using ClueGo, a plug-in developed for Cytoscape [43,44]. ClueGo annotates genes into biological process, molecular function and cellular components. Significantly expressed genes, split as up- or down- expressed, were used as input data for functional analysis. For figures, the *Candida* Genome Database GOSlim annotations, which collapse GO terms into broad, *Candida-*specific categories, were utilized [45]. For tables, the full set of GO terms were used.

## Results

**Fig 1:**
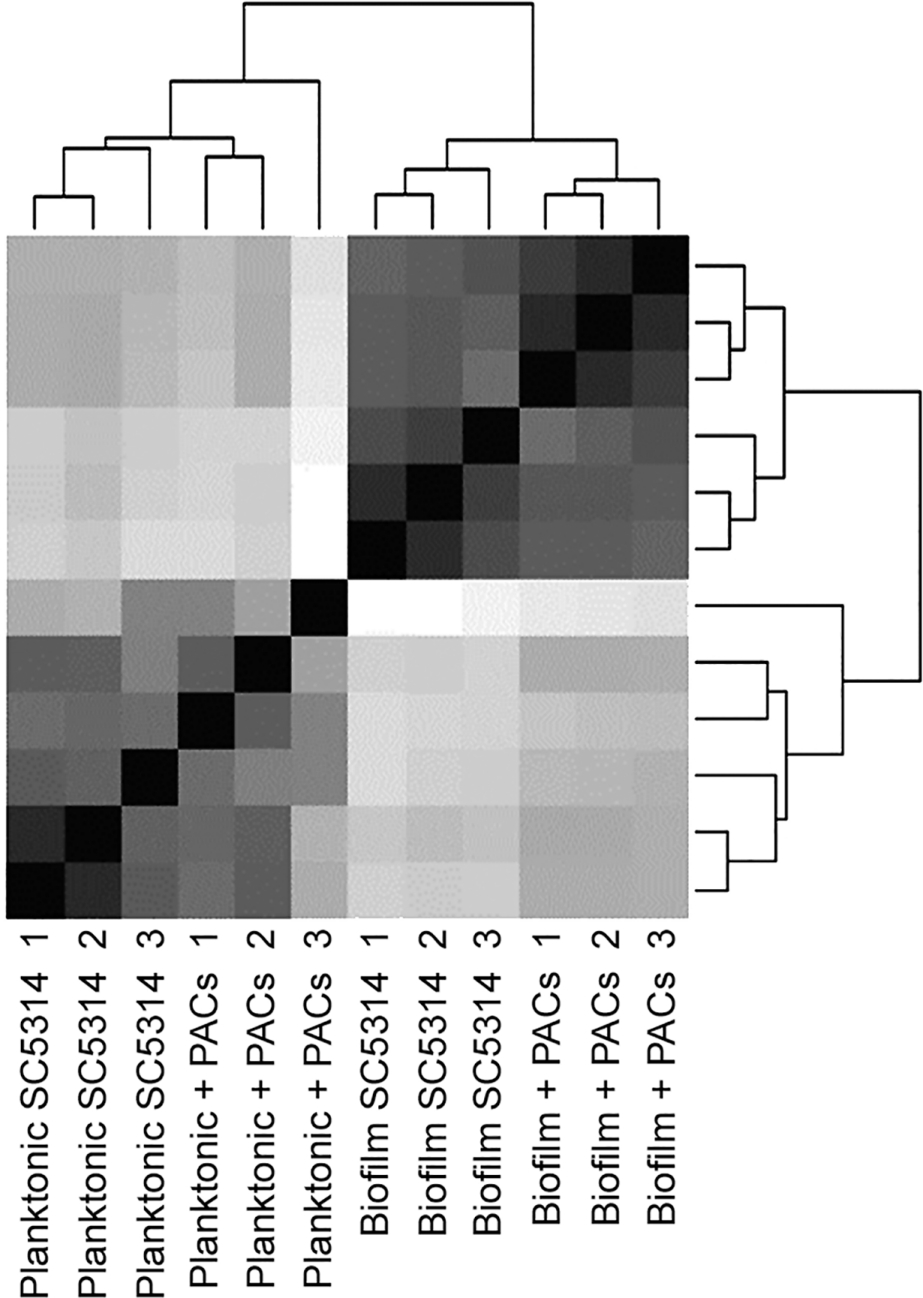
Dendrogram representing hierarchical sample clustering representing high correlation between the samples. Sample clustering was performed as part of the DESeq tool. The dist function was applied to the transpose of transformed count matrix to obtain sample-to-sample distances. A heat map of this distance matrix highlights the differences and similarities between samples.

**Fig 2:**
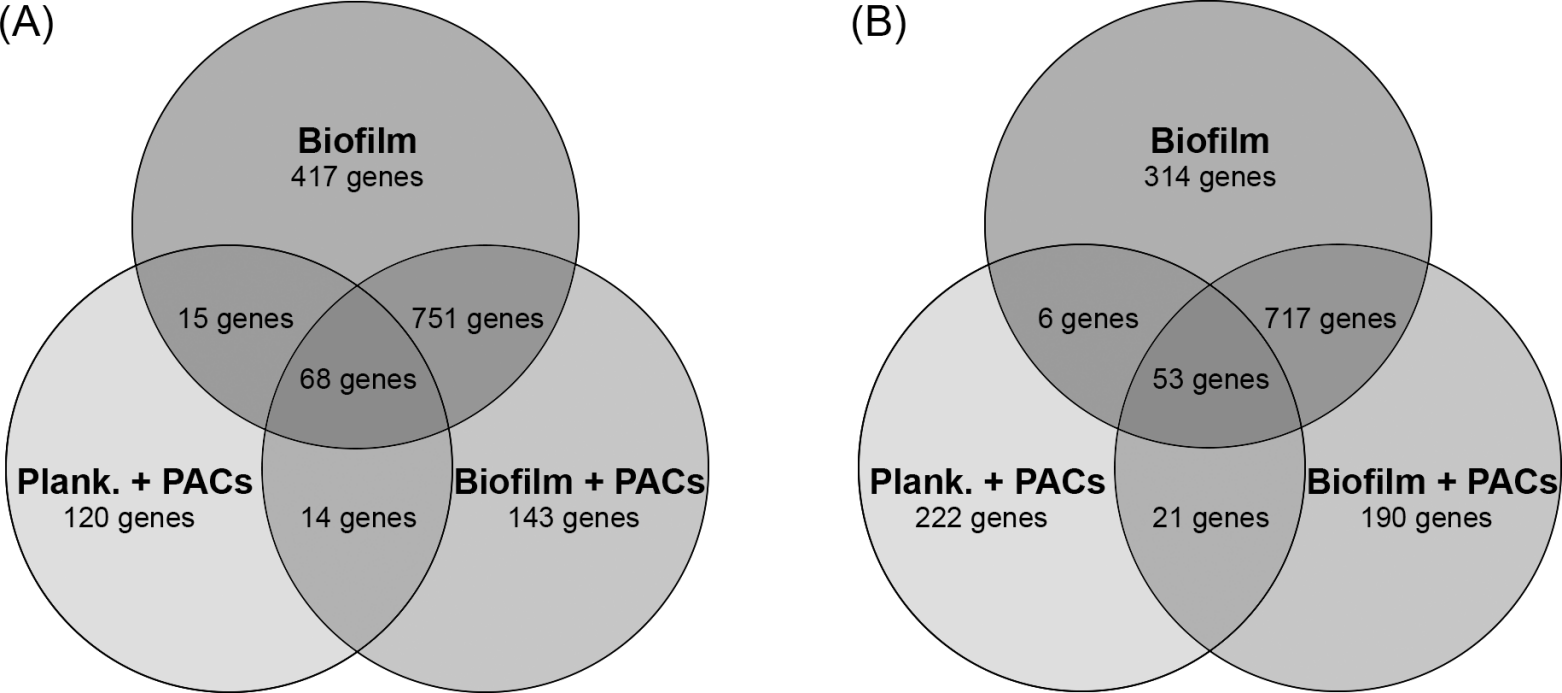
Venn diagram analysis of gene expression. Genes differentially expressed between the three conditions, namely, untreated biofilms, PAC-treated biofilms and PAC-treated planktonic cells were determined as compared to planktonic cells in AU. A) Venn diagram illustrating the number of upregulated genes within each comparison and shared genes between different conditions. B) Venn diagram illustrating the number of downregulated genes within each comparison and shared genes between different conditions.

PACs were isolated from cranberry fruit (*Vaccinium macrocarpon* Ait.) using solid-phase chromatography as described [23]. RNA was isolated after 48h from planktonic and biofilm cells in AU both with and without cranberry PACs. We next used RNA-Seq to profile genome-wide changes in transcription in (i) *C*. *albicans* urinary biofilms compared to planktonic cells, to elucidate functional pathways unique to biofilm development in the artificial urinary environment, and (ii) urinary biofilms treated with PAC compared to untreated urinary biofilms, to elucidate pathways involved in the response to proanthocyanidins. All experiments were completed at the late biofilm stage (48h). Sequencing yielded between 25,152,600 and 30,903,560 high-quality reads per sample (Table 1). High correlation existed between all three replicates within each sample, which was represented as a dendrogram, generated using a hierarchical clustering approach integrated as part of the DESeq tool (Fig 1). A total of 6444 genes had any kind of expression in all conditions, which is a measure of any read aligning to gene features in the Candida Genome Database. Differentially-expressed genes were defined as genes with a fold-change ≥1.5 and a p-value ≤0.05 as compared to planktonic cells grown in AU. Of the genes that were significantly upregulated, 49.1% were common between biofilms with and without PAC treatment (Fig 2A). In contrast, only 0.91% of genes were upregulated in both biofilms and planktonic cells treated with PACs. 9.36% of upregulated genes were unique to PAC-treated biofilms; 7.85% of upregulated genes were unique to PAC-treated planktonic cells; and 27.3% of upregulated genes were unique to biofilms formed in AU without PACs. A similar distribution was observed amongst down-regulated genes (Fig 2B). In both planktonic and biofilm cells treated with PACs, more genes were down-regulated than upregulated.

**Table 1:** Sequencing yield per sample. Four treatments were sequenced using Illumina HiSeq2000 with three replicates each.

Sample	Number of reads per sample
Planktonic SC5314 1	28,614,219
Planktonic SC5314 2	28,079,324
Planktonic SC5314 3	30,903,560
Biofilm SC5314 1	28,474,183
Biofilm SC5314 2	28,146,364
Biofilm SC5314 3	28,598,751
Planktonic + PACs 1	26,913,821
Planktonic + PACs 2	28,634,731
Planktonic + PACs 3	33,308,787
Biofilm + PACs 1	29,288,331
Biofilm + PACs 2	25,152,600
Biofilm + PACs 3	27,178,053

**Fig 3:**
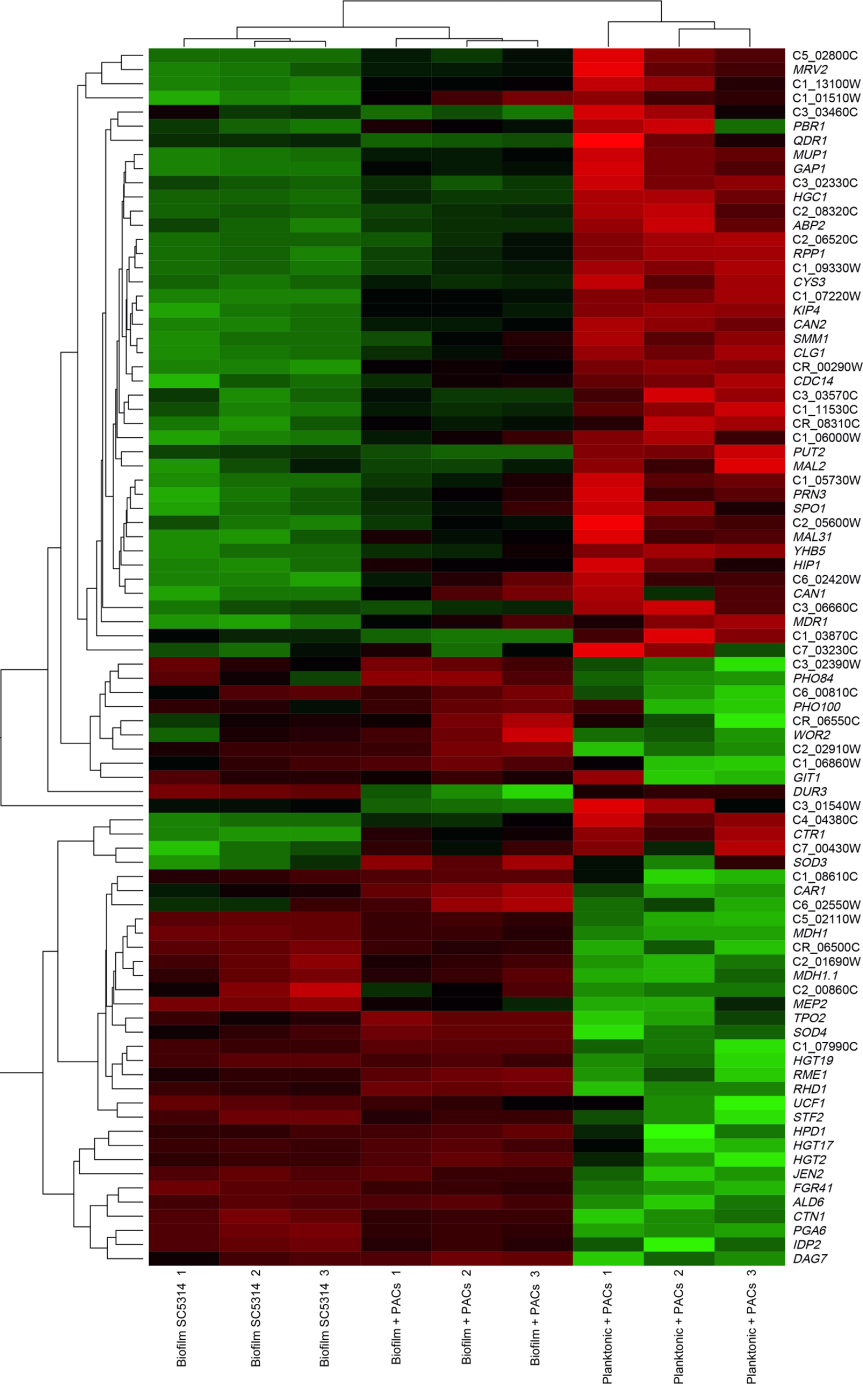
Heatmap of the top differentially-expressed genes across all treatments. The top differentially-expressed genes across all treatments as compared to planktonic cells in AU were selected using DESeq2 with the DEApp tool, with an increased cut-off defined as a p-adjusted value of less than or equal to 0.01 and a fold-change of 5 or higher. The top differentially-expressed genes were visualized using the Heatmapper web tool [42]. Red coloring indicates down-regulation as compared to planktonic cells in AU; black indicates no change in expression compared to planktonic cells in AU; green indicates up-regulation as compared to planktonic cells in AU. Dendrogram indicates hierarchical clustering between samples (top) or genes (right).

The top differentially-expressed genes in all three treatments (biofilm cells, planktonic cells + PACs, and biofilm cells + PACs) compared to planktonic cells in AU are represented via heatmap (Fig 3). Hierarchical clustering revealed that gene expression patterns were more similar between biofilm cells and PAC-treated biofilm cells than between PAC-treated planktonic cells and PAC-treated biofilm cells. This is suggestive of a strong biofilm-specific transcriptional profile; this profile was present but markedly down-regulated upon PAC treatment (Fig 3).

**Fig 4:**
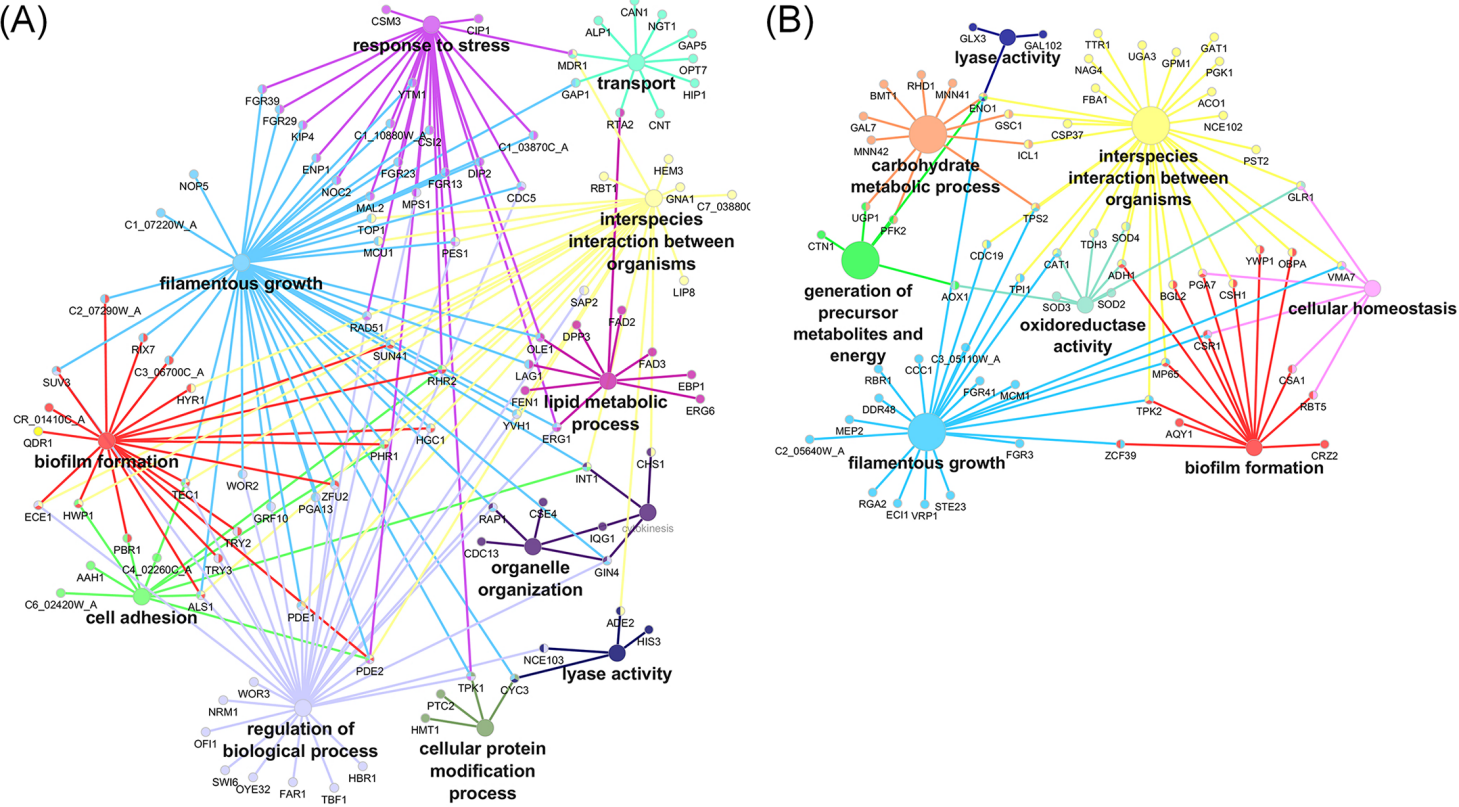
Pathway analysis of differentially-expressed genes as determined by RNA Seq in urinary biofilms. Analysis was completed using the ClueGO plugin for Cytoscape and the *Candida* Genome Database GOSlim annotation. **(A)** Pathway analysis of differentially-expressed genes in urinary biofilms compared to planktonic cells in AU. Genes involved in adhesion, filamentation biofilm formation, transport, lipid metabolism and response to stress were up-regulated in urinary biofilms. **(B)** Genes involved in carbohydrate metabolism, cellular homeostasis, oxidoreductase activity, and lyase activity were down-regulated in urinary biofilms.

**Fig 5:**
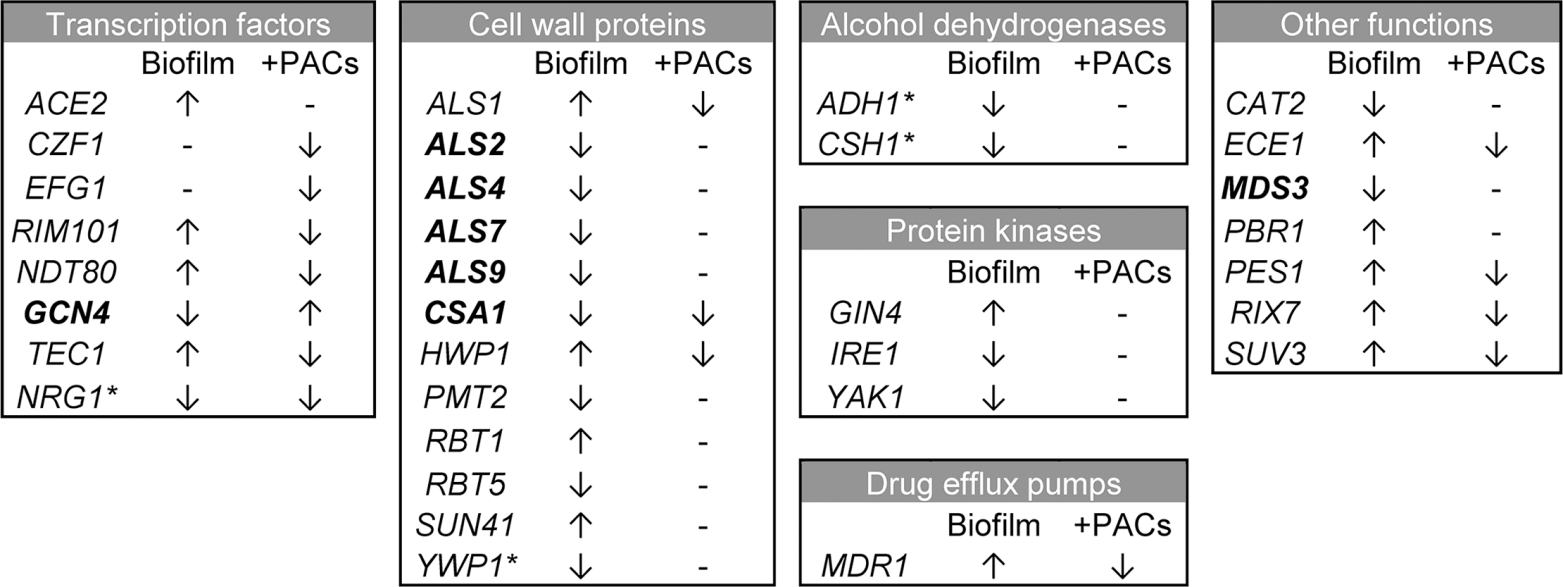
Differentially-expressed biofilm-related genes. Genes previously shown to have roles in biofilm formation were broken into different functional categories as in Finkel and Mitchell [53]. Genes up- or down-regulated in biofilms as compared to planktonic cells or in PAC-treated biofilms as compared to biofilms alone are indicated by arrows. Negative regulators of biofilm formation are indicated by asterisks (*). Genes indicated in bold are those with potentially unexpected expression changes compared to biofilms formed in serum-like conditions.

A total of 2341 out of 6434 genes had significant changes in expression in biofilm cells relative to planktonic cells (S1 and S2 Tables). Functional pathway analysis revealed that genes involved in filamentous growth, adhesion, drug response, stress response, and oxidation-reduction processes were up-regulated in biofilms (Fig 4A, S1 Table). Many of these differentially-expressed genes have previously been shown to play roles in *C*. *albicans* biofilm formation (Fig 5). Several transcription factors involved in hyphal formation were upregulated in our dataset. These included *TEC1*, *ACE2*, *RIM101* and *OFI1* [46–49]. Other upregulated genes with more direct roles in hyphal biology included *HWP1* and *ECE1* [50,51]. Genes such as *PBR1* (unknown function) and *ALS1* (adhesion) have been implicated in biofilm formation and are thought to be involved in the adherence stage of biofilm formation [52–54]. Amino acid transport was the most significantly enriched GO term in this dataset (S1 Table); upregulated amino acid transport genes included *ALP1*, *CAN1*, and the general amino acid permeases *GAP1*, *GAP4*, *GAP5* and *GAP6*. Notably, methionine biosynthesis and phosphate transport pathways are up-expressed in early biofilm formation [55], and methionine biosynthetic pathways remain prominently up-expressed in late stage biofilms [56]. Methionine biosynthetic pathway genes were also up-expressed in late stage urinary biofilms (genes included *MET1*, *MET3*, *MET10*, and *MET15*). In addition, the high-affinity phosphate transporter *PHO84* [57] and the putative phosphate transporter *PHO87* [58] were up-expressed in biofilm compared to planktonic cells. Lipid metabolism genes were also upregulated in urinary biofilms, including genes involved in ergosterol, cholesterol, sphingolipid, and oleic acid synthesis (S1 Table). Among genes that were down-expressed in biofilms relative to planktonic cells, many were involved in endocytosis, vesicle-mediated transport, nitrogen and carbon metabolism, oxidoreductase activity, and nutrient response (Fig 4B, S2 Table). The downregulation of metabolic processes is not surprising as biofilm cells were harvested at 48hr, and metabolic genes are down-regulated in mature biofilms [59,60]. A complete list of enriched GO terms that are down-expressed in biofilms compared to age-matched planktonic cells is provided in S2 Table.

**Fig 6:**
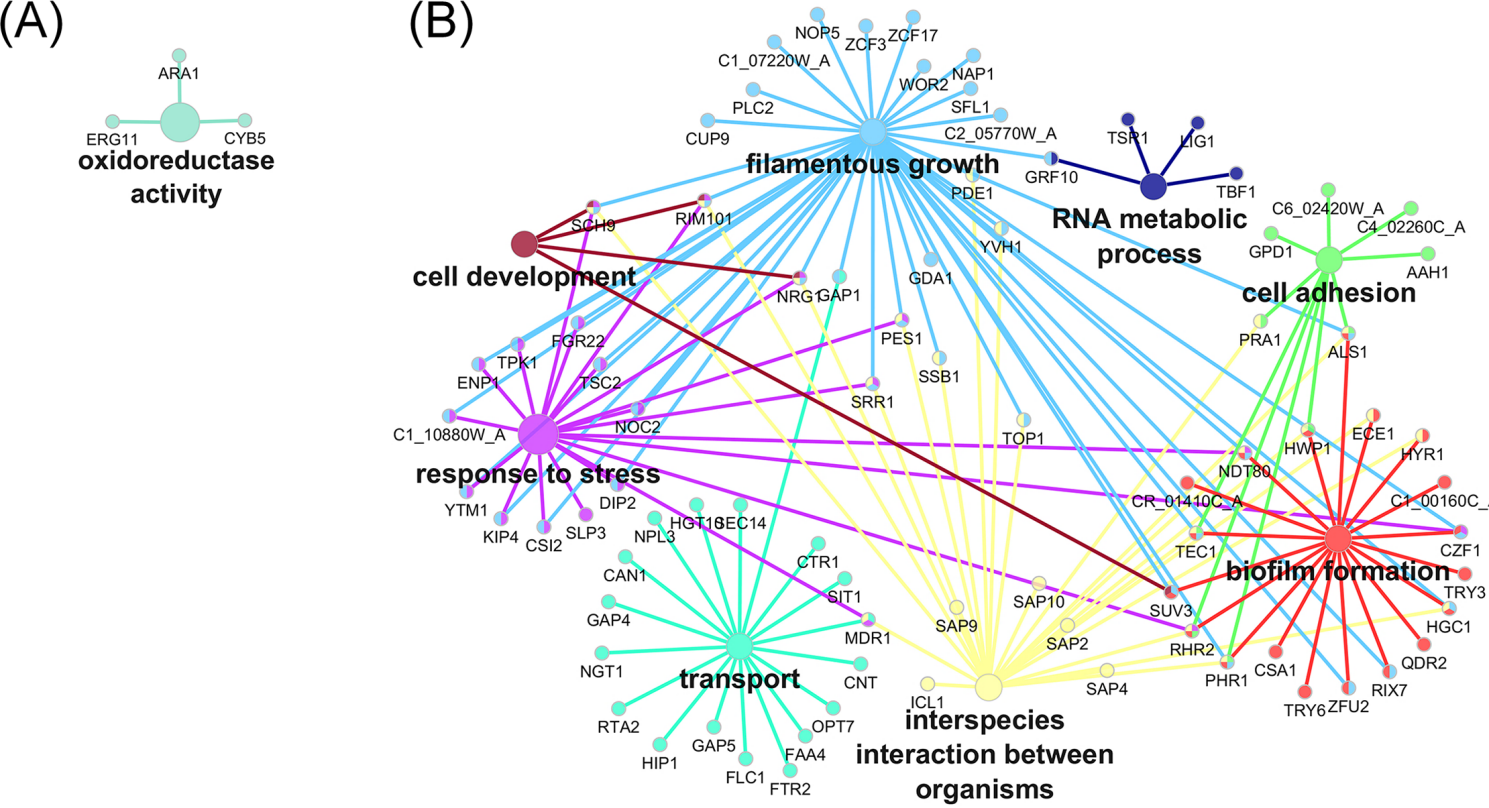
Pathway analysis of differentially-expressed genes as determined by RNA Seq in PAC-treated biofilms. Analysis was completed using the ClueGO plugin for Cytoscape and the *Candida* Genome Database GOSlim annotation. (A) Genes that are up-expressed in PAC-treated biofilms were enriched for oxidoreductase-activity related genes. (B) Genes that are down-expressed in PAC treated biofilms and the genes mainly fall under processes such as regulation of filamentous growth, adhesion and biofilm formation.

When PAC-treated urinary biofilms were compared with untreated urinary biofilms, 556 of 6434 genes had significant changes in gene expression (S4 and S5 Tables). Up-regulated genes in biofilm cells treated with PACs relative to biofilm control cells were involved in oxidoreductase activity, transmembrane transport, and alcohol biosynthesis (Fig 6A, S4 Table). Genes down-regulated were implicated in adhesion, filamentation, biofilm formation, drug transport, and iron metabolism (Fig 6B, S5 Table). Complete gene lists associated with their GO terms are available as supplemental data (S4 and S5 Tables). Specific down-regulated genes included the secreted aspartyl protease *SAP9* (down-regulated five-fold), and the major adhesin *ALS1* (down-regulated three-fold). In addition, *FRE30* and *FRE7* have sequence similarity to ferric reductases [58], and *FET31* and *SIT1* are known iron-starvation related genes [61,62]. Other genes involved in iron regulation included *RIM101* (down-regulated 2.5-fold) which is required for expression of iron uptake genes in neutral or alkaline conditions [63]; *FTR2*, which encodes a high-affinity iron permease [64] whose transcript is induced in low iron [65]*; FLC1*, which encodes an iron-starvation-regulated heme uptake protein [66], and *MRS4*, an iron transporter that mediates Fe^2+^ transport across the inner mitochondrial membrane under low iron conditions [67]. Overall, iron uptake genes are down-regulated upon treatment with PACs. As artificial urine is a low-iron environment, this indicates that PAC treatment inhibits the expression of iron acquisition genes in response to low environmental iron levels. Thus, pathways related to adhesion and to inhibition of adaptations to a low-iron environment are important in the response to PAC inhibition of *C*. *albicans* urinary biofilms. Overall, PACs induced changes in adhesion and iron metabolism pathways within late stage urinary biofilms.

We also analysed gene expression in PAC-treated planktonic cells as compared to untreated planktonic cells to differentiate between PAC response in planktonic cells versus biofilms (S6–S9 Tables). Like PAC-treated biofilms, genes downregulated in PAC-treated planktonic cells were enriched for genes involved in iron homeostasis (S7 Table). Several of the specific genes down-regulated in PAC-treated biofilms were also down-regulated in PAC-treated planktonic cells; these genes included *FET31*, *FRE7*, *FRE30*, *CTR1*, and the secreted aspartyl proteases *SAP4* and *SAP5* (S9 Table). Interestingly, the number of differentially-expressed genes shared between PAC-treated biofilms and PAC-treated planktonic cells was low (14 upregulated genes and 21 downregulated genes; see Fig 2). Thus, cranberry PACs induce distinct transcriptomic landscapes in biofilms and planktonic cells.

## Discussion

The global transcriptional changes involved in the transition of *C*. *albicans* from planktonic growth to biofilms in bloodstream-like conditions have been studied in detail [55,56,68]. In contrast, the transcriptional changes regulating urinary biofilm development are largely unknown, although clear differences in urinary biofilm structure are evident compared to biofilms in bloodstream-like conditions [69]. Overall, comparatively little is known about *C*. *albicans* urinary pathogenesis in general. In these studies, we determined that urinary biofilm formation shares core pathways with biofilms formed under other environmental conditions, including several master transcription factors [68] as well as genes involved in the critical processes of adhesion, filamentation, maturation and dispersal [53]. Genes involved in the process of filamentation, a key component of *C*. *albicans* pathogenesis, including well-described transcription factors involved in hyphal formation, were up-expressed. Adhesion related genes were also up-expressed in urinary biofilms. Additional pathways common to serum-like and urinary biofilms included those involved in amino acid transport, including methionine biosynthesis which is well-described in biofilm formation. Phosphate transporter was also up-expressed. Down-expressed pathways in urinary biofilms included various metabolic pathways and house-keeping functions, which is consistent with the relatively quiescent state of cells within biofilms. However, much of the gene expression patterns in urinary biofilms were not observed under other conditions; this subset of genes included a large number of genes of unknown function.

To delve further into understanding genes and pathways unique to urinary biofilms, we used annotations from the *Candida* Genome Database (CGD) to compare our dataset with previous biofilm studies, including gene expression data from two previous studies of mature biofilms. Nobile et al. (2012) analysed differential gene expression in 48h biofilms formed in Spider medium as compared to planktonic cells [68]. 275 upregulated genes and 148 downregulated genes were shared between biofilms formed in Spider media, and biofilms formed in AU in this study (S3 Table). Of note, Nobile et al. described six master transcriptional regulators, *BCR1*, *BRG1*, *EFG1*, *NDT80*, *ROB1* and *TEC1*, of *C*. *albicans* biofilm formation. Of these, only *NDT80* and *TEC1* were upregulated in late urinary biofilms in this study. Nett et al. (2009) compared expression of mature *in vivo* rat catheter biofilms to *in vitro* planktonic cells [70]. 171 upregulated genes and 105 downregulated genes were shared between rat catheter biofilms and our urinary biofilms (data not shown). Of these, 84 upregulated genes and 34 downregulated genes were common to all three studies, potentially indicating a core set of genes involved in biofilm formation across varying environmental conditions (S3 Table). These shared genes included *ECE1*, *TEC1*, and *ACE2*. We further excluded any genes previously annotated in CGD with roles in biofilm formation. Many differentially-expressed genes in our dataset have not been previously implicated in biofilm formation, indicating a gene expression pattern unique to mature biofilms formed in artificial urine (824 uniquely upregulated genes and 874 uniquely downregulated genes, S3 Table). Functional pathway analysis revealed that these genes were not significantly enriched for any gene ontology terms, with a full 30.3% of genes corresponding to genes of unknown function. Some of these genes may have roles specific to the ecological niche of the urinary tract, an understudied area in *C*. *albicans* commensalism and pathogenesis. The top upregulated gene in this urinary biofilm-specific dataset was *OFI1* (log2FC = 5.42), a putative transcription factor previously shown to be involved in hyphal morphogenesis [68]. These genes of unknown function appear to be unique to urinary biofilm formation and are consequently of great interest for further study. The extensive differences observed between gene expression in urinary biofilms compared to previously studied biofilms are likely due to environmental differences, including nutrient availability and external pH. Likewise, there have been no previous studies of the transcriptomic response of *C*. *albicans* biofilms to cranberry-derived PACs. In previous work, we demonstrated that cranberry PACs inhibit urinary biofilm formation, but have minimal effects on planktonic cell growth [31]. We also assayed expression of selected key adhesin genes within urinary biofilms subjected to PACs in early urinary biofilms via qRT-PCR and discovered biofilm-induced gene expression changes (unpublished data). Based upon these preliminary observations, we then used RNA-Seq to study the global transcriptional response of late biofilms subjected to PAC inhibition, and found that PAC treatment induced down-regulation of genes involved in iron uptake, adhesion, filamentation, and biofilm formation. Notable biofilm-related genes that were down-expressed included the secreted aspartyl protease *SAP9*, and the adhesin *ALS1*. Prominently, a number of genes related to iron starvation and iron regulation were down-regulated in urinary biofilms exposed to PACs. Taken together, cranberry-derived PACs inhibit response to low iron, adhesion, and multiple key biofilm-specific processes. Previously, we showed that addition of exogenous iron to AU biofilms partially restores *C*. *albicans* biofilm-forming capacity [31]. These transcriptomic data indicate that cranberry PACs interfere with *C*. *albicans* tolerance of low-iron, but not iron-replete, environments.

It is quite interesting that cranberry-derived PACs inhibit both *E*. *coli* and *C*. *albicans* adherence and iron acquisition *in vitro*. PACs can be expected to chelate exogenous iron, thus impacting iron availability and consequently iron metabolism in any organism. It is difficult to say whether organism-specific mechanisms also contribute, though study of various *C*. *albicans* iron uptake mutants may help further elucidate the responsible mechanisms. Interference of non-specific adhesion could also be expected to occur regardless of the organism. However, the explanation for shared adhesion mechanisms across kingdoms is not immediately intuitive. We speculate that over the course of host-pathogen evolution, mechanisms of pathogen adherence to host substrates have evolved convergently. Thus, although well beyond the scope of this study to investigate, we suspect that cross-kingdom inhibition of adherence by PACs is due to common evolutionary demands, such that distinct adhesion molecules have converged enough in structure and function to allow PAC inhibition of adherence even in organisms that evolved pathogenicity independently.

The overall goal of these experiments is to develop a novel strategy for prevention of *Candida* UTIs. Whether this involves different types of PACs, more potent PAC derivatives, or PACs in combination with other preventative agents or strategies remains to be determined. From a mechanistic standpoint, further studies incorporating genetic mutants of genes in pathways of potential importance to *C*. *albicans* urinary biofilm formation could be studied in relation to the effect of PACs. Further, we intend to use dual RNA-Seq [71] to simultaneously investigate the pathogen and mammalian cell transcriptomes during simulated *C*. *albicans* urinary infection. These studies will include comparisons in conditions including PACs to further elucidate the biological pathways relevant to *Candida* UTIs and PAC prevention. Next, studies using animal models of urinary infection could be used to confirm the efficacy of cranberry-derived PACs in prevention of catheter-associated UTIs due to *C*. *albicans* and other *Candida* species. Finally, we hope to study cranberry-derived PACs for prevention of *Candida* UTIs in catheterized patients. Thus, by understanding the mechanisms of activity of cranberry-derived PACs, we hope to develop PACs or PAC derivatives as a novel therapeutic strategy for the prevention of catheter-associated *Candida* UTIs. Taken together, these studies suggest that cranberry-derived PACs may have promise as a clinically viable therapeutic option to prevent development of fungal urinary biofilms, particularly in patients with indwelling urinary catheters.

## Supporting information

S1 Table Genes up-expressed in WT biofilm cells relative to WT planktonic cells. Sheet 1 contains gene expression information. Sheet 2 contains GO term enrichment analysis.
(XLSX)

S2 Table Genes-down expressed in WT biofilm cells relative to WT planktonic cells. Sheet 1 contains gene expression information. Sheet 2 contains GO term enrichment analysis.
(XLSX)

S3 Table Comparison of differentially-expressed genes with published studies. (XLSM)

S4 Table Genes up expressed in PAC-treated biofilm cells relative to biofilm control cells. Sheet 1 contains gene expression information. Sheet 2 contains GO term enrichment analysis.
(XLSX)

S5 Table Genes down expressed in PAC-treated biofilm cells relative to biofilm control cells. Sheet 1 contains gene expression information. Sheet 2 contains GO term enrichment analysis.
(XLSX)

S6 Table Genes up-expressed in PAC-treated planktonic cells relative to WT planktonic cells. Sheet 1 contains gene expression information. Sheet 2 contains GO term enrichment analysis.
(XLSX)

S7 Table Genes down-expressed in PAC-treated planktonic cells relative to WT planktonic cells. Sheet 1 contains gene expression information. Sheet 2 contains GO term enrichment analysis.
(XLSX)

S8 Table Genes up-expressed in PAC-treated biofilm cells relative to WT planktonic cells. Sheet 1 contains gene expression information. Sheet 2 contains upregulated genes shared between PAC-treated planktonic cells and PAC-treated biofilm cells relative to WT planktonic cells, and GO term enrichment analysis of this data.
(XLSX)

S9 Table Genes down-expressed in PAC-treated biofilm cells relative to WT planktonic cells. Sheet 1 contains gene expression information. Sheet 2 contains downregulated genes shared between PAC-treated planktonic cells and PAC-treated biofilm cells relative to WT planktonic cells, and GO term enrichment analysis of this data.
(XLSX)
